# The first report of seroprevalence of Q fever in water buffaloes (*Bubalus bubalis*) in Phatthalung, Thailand

**DOI:** 10.14202/vetworld.2021.2574-2578

**Published:** 2021-09-29

**Authors:** Kamchai Kidsin, Decha Panjai, Sumalee Boonmar

**Affiliations:** 1 Animal Health Section, The Eight Regional Livestock Development, Muang Surat Thani, Surat Thani Province, 84000 Thailand; 2 National Institute of Health, Department of Medical Sciences, Ministry of Public Health, Nonthaburi, 11000, Thailand; 3 Akkhraratchakumari Veterinary College, Walailak University, 222 Thaiburi, Thasala, Nakhon Si Thammarat 80160, Thailand; 4 One Health Research Center, Walailak University, Nakhon Si Thammarat 80160, Thailand.

**Keywords:** *Coxiella burnetii*, indirect immunofluorescence assay, Q fever, seroprevalence, Thailand, water buffaloes

## Abstract

**Background and Aim:**

Q fever is a worldwide zoonosis caused by the intracellular bacterium, *Coxiella burnetii*. A few studies focused on the occurrence of Q fever infection in water buffaloes in Thailand have been conducted; however, little is known regarding the seroprevalence of *C. burnetii* antibodies in buffaloes. In the present study, we describe the prevalence of Q fever infection in water buffaloes (*Bubalus bubalis*) in Phatthalung, Thailand.

**Materials and Methods:**

A total of 421 samples (156 blood, 156 sera, and 109 ectoparasites [lice]) were collected from 156 water buffaloes from 29 farms of the Phatthalung Province from January 22, 2021, to March 26, 2021. The blood and ectoparasite samples were screened for *C. burnetii* DNA using a polymerase chain reaction assay and the sera were tested for *C. burnetii* antibody using an indirect immunofluorescence assay.

**Results:**

*C. burnetii* DNA was not detected in blood or ectoparasites; however, the seroprevalence of individual water buffaloes was 4.49% (95% CI: 2.19-8.99%), whereas that of the herd was 13.79%. There was a significant difference between abortion history and Q fever infection at 29 farms (p=0.005; OR=33.55 [95%CI: 156-722.38]).

**Conclusion:**

This is the first report describing the low seroprevalence of *C. burnetii* antibodies in water buffaloes in Phatthalung Province, Thailand. The occurrence of this pathogen in buffaloes with reproductive disorders and people working with buffaloes warrant further investigation. Animal health authorities should inform farmers to effectively prevent and control this zoonosis.

## Introduction

Q fever is a global zoonotic disease caused by *Coxiella burnetii*, a small obligate intracellular Gram-negative bacterium [1]. This pathogen can infect humans and animals through inhalation of dust contaminated with birth products, tick feces, or placenta secretions. Other routes of transmission include foodborne infection or contact with reservoir ectoparasites [1]. Animals are often sub-clinically infected and may present with reproductive disorders, such as abortion, premature delivery, or stillbirth. This infection is also of economic significance in farm animals, especially cattle, water buffaloes, sheep, and goats. Water buffaloes (*Bubalus bubalis*) belong to the *Bovidae* family, tribe *Bovini*, which is a subtribe of wild cattle. Along with cattle, these animals are an important agricultural and economical source of milk, meat, draught power, and manure-based fertilizer. Therefore, infection has the potential to cause significant losses to the livestock industry. In Thailand 2020, a total of 1,256,074 water buffaloes were maintained (data report from Information and Communication Technology Center, Department of Livestock Development).

Q fever in Thailand has been studied since 1966 [2]. Several studies have demonstrated the prevalence and seroprevalence of this pathogen in humans and animals [3-6]. However, few studies of the occurrence of Q fever infection in water buffaloes have been conducted [7-10]. Nevertheless, there was only one report in Thailand focused on *C. burnetii* DNA from the placenta of water buffaloes [11]. In addition, the seroprevalence of *C. burnetii* antibody in buffaloes has not been established. Several tests are available to detect *C. burnetii* antibody from human and animal samples, including enzyme-linked immunosorbent assay (ELISA) [8,12] and the Indirect Fluorescent Assay (IFA) [13-15]. Recently, IFA has been shown to detect both Phase I (virulent C1) and Phase II (non-virulent C2) antibodies. In 2012, the National Institute of Health (NIH) Thailand developed an IFA for Q fever diagnosis using an antigen provided by the US-CDC.

In the present study, we describe the prevalence of Q fever infection in water buffaloes (*B. bubalis*) in Phatthalung, Thailand. These results provide insight for researchers and the public health authority for the development of methods to prevent and control this disease.

## Materials and Methods

### Ethical approval

The collection procedures in the animal have been approved by the Institutional Animal Care and Use Committee of the NIH Thailand.

### Study period and area

**Figure-1:**
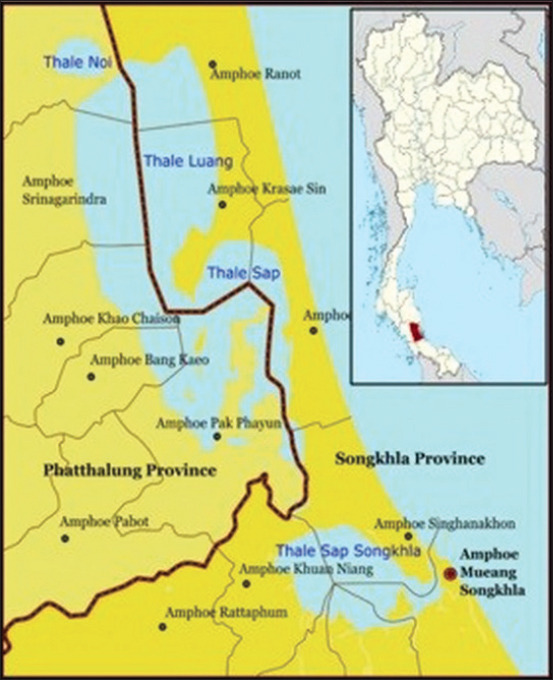
Map of study area, Thale Noi, Phatthalung Province, Southern part of Thailand [Source: https://images.app.goo.gl/2x8P77H3JctVhXny5].

The study was conducted from 1 January to 31 May 2021. The present study place was Thale Noi in the Phatthalung Province in the southern part of Thailand located at 7 ͦ46 ′ 00 ′ N 100 ͦ09 ′ 11 ″ E (Figure-1) where over 3000 water buffaloes are raised.

### Sample collection

A total of 4124 water buffaloes from 356 farms in Phatthalung province were analyzed using data from the Information and Communication Technology Center, Department of Livestock Development. We calculated a sample size from the population of 3,000 water buffaloes at Thale Noi using the Epitools program (www.epitool.net) with a 95% CI and 2.5% precision. The results indicated that a sample size of 169 specimens was required. From January 22, 2021, to March 26, 2021, 421 samples including 156 blood, 156 sera, and 109 ectoparasites (lice) were collected from 156 water buffaloes at 29 farms. Sample collection ended because of the COVID -19 pandemic after March 2021. All blood and sera were collected from each animal aseptically into 10 mL tubes containing ethylenediaminetetraacetic acid tubes and 5 mL of plain tubes. Ectoparasites were kept in sterile glass vials. The history of each water buffalo was recorded with respect to sex, age, and health status. The samples were transported to the Department of Medical Sciences at the NIH laboratory under chilled conditions and stored at −20°C until further processing.

### DNA extraction from blood and ectoparasites and polymerase chain reaction (PCR) assay

DNA was extracted from the blood and ectoparasite samples using the DNeasy blood and tissue kit (250) from Qiagen (Hilden, Germany) according to the manufacturer’s instructions. *C. burnetii* DNA was assessed by PCR using the conditions as previously described [16]. Briefly, the DNA extraction product and two specific primer sets (Cox 2 primer pair: 5’-CAACCCTGAATACCCAAGGA-3’ and 5’- GAAGCTTCTGATAGGCGGGA-3’ and Cox 5 primer pair: 5’-CAGGAGCAAGCTTGAATGCG-3’ and 5’- TGGTATGACAACCCGTCATG -3’) were placed into a PCR reaction (initial denaturation of 10 min at 95°C, followed by 37 cycles of denaturation for 30 s at 95°C, annealing for 30 s at 57°C, and extension for 1 min at 72°C) and mixed with PCR master mix reagent. The PCR products of 397 (Cox2) and 395 (Cox5) base pairs were obtained from positive samples.

### Standard strains of *C. burnetii*

C Phase I is virulent phase I (LPS I) of *C. burnetii* and C PHASE II is avirulent Phase II (LPS II) of *C. burnetii*. Both strains were kindly provided by the US-CDC.

### Indirect immunofluorescence assay (IFA)

Briefly, each serum sample (20 mL) was diluted 2-fold with 40 mL of 1× PBS. The reference antigens (C Phase I and C Phase II) were coated onto an immunofluorescent glass slide and the serum samples containing immunoglobulin were fixed with the coated antigen. After a washing step, a fluorescent-labeled anti-serum immunoglobulin was added. The slides were then examined by fluorescence microscopy. A positive result was defined as a fluorescent appearance and the laboratory results were interpreted using acceptable guidelines. Buffalo sera exhibiting an antibody titer equal or greater than 16 were considered positive.

### Statistical analysis

Fisher’s exact test and odds ratio were used in a univariate analysis. p≤0.005 was considered statistically significant.

## Results

We collected 156 blood, 156 sera, and 109 ectoparasites (lice) from 29 farms at Thalae Noi, Phatthalung Province. The results indicated that 7 of 156 buffaloes (4.49%, 95% CI: 2.19-8.97%) were seropositive for *C. burnetii* infection, although *C. burnetii* DNA was not detected in any of the buffaloes (Table-1).

**Table-1:** Seroprevalence of *Coxiella burnetii* antibody in buffaloes sera by IFA and detection of *Coxiella burnetii* DNA from blood and ectoparasite samples by polymerase chain reaction.

Seroprevalence			Detection of *Coxiella burnetii* DNA		
	
Serum	No. of positive	Percentage	Blood/ectoparasite	No. of positive	Percentage
156	7	4.49	156/109	0	0

The herd seroprevalence was 13.79% (4/29). Of 29 farms, seven positive buffaloes were detected at four farms. Farm 2 consisted of two buffaloes, each containing 1:32 C Phase I and 1:16 C Phase II antibody. Farm 3 consisted of three buffaloes containing 1:32 C Phase I, 1:16 C Phase II, and 1:16 C Phase II, respectively. Farm 4 and farm 8 consisted of one buffalo each containing 1:16 C Phase I and 1:256 C Phase II, respectively (Table-2). Moreover, there was a significant difference between abortion and Q fever infection from 29 farms (p=0.005, OR=33.55 (95%CI: 1.56-722.38]) (Table-3). Four of 29 farms contained seropositive buffaloes with an abortion history">1.56-722.38]) (Table-3). Four of 29 farms contained seropositive buffaloes with an abortion history, whereas we did not find seropositive at the other 25 farms; however, five out of 25 farms had an abortion history.

**Table-2:** Herd seroprevalence and titer of C Phase I and C Phase II in positive buffaloes.

Farm	No. of buffaloes	No. of positive buffaloes (%)	Titer of C phase I	Titer of C phase II
1	11	-	-	-
2*	6	2 (33.33)	1:32	1:16
3*	6	3 (50)	1:32	1:16, 1:16
4*	14	1 (7.14)	1:16	-
5	11	-	-	-
6	12	-	-	-
7	12	-	-	-
8*	12	1 (8.33)	1:256	-
9	9	-	-	-
10	7	-	-	-
11	4	-	-	-
12	8	-	-	-
13	2	-	-	-
14	1	-	-	-
15	2	-	-	-
16	2	-	-	-
17	2	-	-	-
18	1	-	-	-
19	2	-	-	-
20	2	-	-	-
21	1	-	-	-
22	1	-	-	-
23	1	-	-	-
24	9	-	-	-
25	3	-	-	-
26	3	-	-	-
27	6	-	-	-
28	4	-	-	-
29	2	-	-	-

* Positive farms containing seropositive antibody water buffaloes

**Table-3:** The association between abortion history and Q fever infection from 29 farms.

Detection by IFA	Abortion history		Total

	Y	N	
Seropositive	4	0	4
Seronegative	5	20	25
Total farm	9	20	29

## Discussion

The seroprevalence of *C. burnetii* antibody was illustrated for the 1^st^ time in water buffaloes from Phatthalung Province in Thailand. Seven buffaloes from four seropositive farms were found to contain *C. burnetii* antibodies. The seroprevalence of individual buffaloes was 4.49% (7/156), whereas that of the herd level was 13.79% (4/29). A low seroprevalence was obtained using IFA; moreover, no *C. burnetii* DNA was identified in the ectoparasites. The previous study[15] indicates that the range of seroprevalence in ruminants depends on the serological methods and the cutoff value used as well as the geographical location and type of animal husbandry. The seropositivity in buffaloes with reproductive disorders in India was 15.15% (5/33) as determined by IFA [7]. Similarly, to another study indicated that the seroprevalence of buffaloes with reproductive disorders in Egypt was 11.2% using ELISA [8]. However, our IFA results corresponded with that of another report from India [9] indicating 3.9% (9/232) seropositivity from healthy buffaloes using ELISA.

Serological techniques only indicate exposure of animals to *C. burnetii* and not the presence of the organism, whereas PCR can reveal the shedding status of the animal by detecting DNA. It should be noted that our samples were collected from healthy water buffaloes and revealed that no *C. burnetii* DNA was present in blood or the ectoparasites. The reason for this was the absence of DNA and a low titer of antibody in these animals.

The previous studies showed that this pathogen can cause problems in the reproductive system of ruminants [17,18]. DNA was detected in 15.15% (5/33) of the buffaloes in India with reproductive disorders [7]. In contrast, only 2.8% (3/108) of healthy water buffalo blood and 2.4% (5/206) from ticks in the Philippines were positive for *C. burnetii* DNA [10]. We did not detect any *C. burnetii* DNA in healthy buffaloes, which is consistent with previous reports regarding the prevalence of DNA in normal buffalo placentas in the northeast and northern region of Thailand, which was only 2.75% (22/800) [11]. These results indicated a low prevalence of *C. burnetii* and the presence of DNA in contaminated bedding, manure, and the surrounding environment. In the present study, we found a significant difference between Q fever and abortion; however, a previous report [19] described no association of Q fever with abortion.

Q fever is an example of a “One Health” approach. There are several studies also describing the occurrence of this infection in people working with ruminants, such as farmers, veterinarians, and animal health officers [3,6,20,21]. Unfortunately, we did not collect human samples for analysis in the present study. Further serological studies should be done using specimens from farmers and those working closely with water buffaloes. Thus, a “One Health” strategy for engaging humans, animals, and environmental professionals is needed to prevent and control *C. burnetii* infection.

## Conclusion

This is the first report of the low seroprevalence of *C. burnetii* antibodies in water buffaloes in Phatthalung Province, Thailand. Further epidemiological studies should be done to understand the risk factors of Q fever in buffaloes and humans.

## Authors’ Contributions

KK: Collected samples and performed experiments. DP: Provided technical help during the experiments. SB: Designed the experiments and revised the manuscript. All authors read and approved the final manuscript.
